# Snowglow—The Amplification of Skyglow by Snow and Clouds Can Exceed Full Moon Illuminance in Suburban Areas

**DOI:** 10.3390/jimaging5080069

**Published:** 2019-08-01

**Authors:** Andreas Jechow, Franz Hölker

**Affiliations:** 1 Ecohydrology, Leibniz-Institute of Freshwater Ecology and Inland Fisheries, 12587 Berlin, Germany; 2 Remote Sensing, GFZ German Research Centre for Geosciences, 14473 Potsdam, Germany; 3 Institute of Biology, Freie Universität Berlin, 14195 Berlin, Germany

**Keywords:** light pollution, artificial light at night, night-time, snow, winter

## Abstract

Artificial skyglow, the fraction of artificial light at night that is emitted upwards from Earth and subsequently scattered back within the atmosphere, depends on atmospheric conditions but also on the ground albedo. One effect that has not gained much attention so far is the amplification of skyglow by snow, particularly in combination with clouds. Snow, however, has a very high albedo and can become important when the direct upward emission is reduced when using shielded luminaires. In this work, first results of skyglow amplification by fresh snow and clouds measured with all-sky photometry in a suburban area are presented. Amplification factors for the zenith luminance of 188 for snow and clouds in combination and 33 for snow alone were found at this site. The maximum zenith luminance of nearly 250 mcd/m^2^ measured with snow and clouds is a factor of 1000 higher than the commonly used clear sky reference of 0.25 mcd/m^2^. Compared with our darkest zenith luminance of 0.07 mcd/m^2^ measured for overcast conditions in a very remote area, this leads to an overall amplification factor of ca. 3500. Horizontal illuminance measurements show values of up to 0.79 lx, exceeding maximum possible full-moon illuminance levels by more than a factor of two. Additional measurements near the Arctic Circle for clear and overcast conditions are presented and strategies for further studies are discussed. We propose the term “snowglow” to describe the amplification of skyglow by snow with and without clouds.

## 1. Introduction

Artificial light at night (ALAN) is a worldwide environmental problem affecting a majority of the world’s population [1] and it is exponentially growing in radiance and extent [2]. The resulting light pollution, first identified as a problem by astronomers [3], can have diverse impacts on flora and fauna [4,5] and potentially human health [6]. Ecological light pollution [5], for example, has impacts ranging from plants [7] to individual animal species on all scales—from micro-organisms [8] to mammals [9], from terrestrial to aquatic habitats [10], and from disturbance of ecosystem services [11] to changed daytime behavior [10].

Artificial skyglow is a form of light pollution that describes the part of ALAN that is emitted upwards, scattered within the atmosphere and diverted back to the Earth’s surface [1,12]. Skyglow is very dynamic as it depends on weather-phenomena like clouds [13,14,15,16,17,18] or on the switching on and off of ALAN sources [19,20]. In an urban context, clouds amplify skyglow [13,14], while in a natural setting clouds should darken the sky [15,16,17,18]. A common way to reduce skyglow or astronomical light pollution in general is to avoid ALAN that is directly emitted upwards, for example by shielded luminaires [21]. However, a fraction of the light emitted downwards will be reflected or scattered at surfaces like the ground or, for example, building walls [12], and therefore, the amount of ALAN contributing to skyglow also depends on the reflectance of the surface material [22]. Snow has a very high albedo, or reflectance over a broad range in the visible spectrum [23]. Thus, snow can dramatically amplify skyglow depending on the original ground albedo [22]. Unfortunately, very few studies on the topic exist [22,23,24,25,26]. Falchi analyzed several datasets in the Mediterranean, mainly Italy and could estimate the amount of uplight by local lamps [21]. Kubala et al. found an increase in night sky brightness from 19 mag/arcsec^2^ to 17 mag/arcsec^2^ with percentage of snow cover determined with a Sky Quality Meter (SQM) near Cracow, Poland [23], and Kolláth determined an increase measuring the night sky brightness also with an SQM as a function of distance from a town in Hungary [24]. Posch et al. analyzed a large multi-year SQM dataset in Upper Austria and also discussed the potential brightening at zenith in winter due to the presence of snow [26].

While SQM measurements are good for long-term monitoring [27], the devices have several drawbacks. SQMs measure the night sky brightness at zenith in a small angle and thus, illuminance cannot be determined with them. Furthermore, they do not provide any color information, which is crucial when changing from classical light sources to solid-state lighting. We therefore prefer photometry with DSLR cameras with fisheye lenses because it provides multi-spectral, spatially resolved radiance information over the whole hemisphere and allows to extract illuminance and color information. Please see more discussion in a review [27] and a method paper [28].

In this paper, all-sky differential photometry [18] is used to track the changes in skyglow, night sky brightness and illuminance with the presence of snow and clouds. A dramatic increase of skyglow with snow and clouds is observed at a suburban site. Amplification factors reach more than 180 and horizontal illuminance levels exceed that of the maximum possible full moon by more than a factor of 2 [29]. The term “snowglow” is introduced for the amplification of skyglow by snow.

## 2. Materials and Methods

### 2.1. Camera Measurement System and Processing Software

A commercial digital camera (Canon EOS 6D, Canon Inc., Tokyo, Japan) with a circular fisheye lens with 180° field of view (Sigma EX DG, f = 8 mm, aperture 3.5) was mounted on a tripod. Auxiliary equipment included a remote control, heating pads to avoid dew or ice formation on the lens, and a bull’s eye spirit level to align the camera with respect to the horizon. To acquire all-sky images, most relevant for astronomical light pollution, the camera was aligned with the imaging sensor in the horizontal plane, i.e., the lens pointing towards the zenith. The full procedure including how to align (in all-sky and vertical plane), focus, and find the right setting for shutter speed and ISO is described in detail in a method paper [28]. For a near-natural sky, the EOS 6D is operated at ISO 3200 or 6400, and up to 120 s exposure time. In urban environments, ISO 1600 and exposure times as short as a 0.4 s are used. Aperture is always set to maximum of 3.5. Images are stored in an unaltered raw format.

For processing the images, the commercial “Sky Quality Camera” (SQC) software (latest version 1.8.1, Euromix, Ljubljana, Slovenia) is used, calculating luminance using the green channel of the camera (that matches photopic vision relatively closely). Photometric calibration is done based on classical astronomical photometry using star brightness and extinction measurements during a night of photometric quality (i.e., stable atmospheric conditions) [27]. Lens vignetting is corrected in the laboratory [30]. The SQC software processes the luminance $L_{v,sky}$ of the sky for each pixel of the camera (luminance is commonly referred to as “brightness” referenced to human vision). From the spatially resolved luminance maps, the software can derive the cosine corrected illuminance $E_{v,cos}$ in the imaging plane:

(1) $$E_{v,cos}=\int_{0}^{\frac{\pi }{2}}\int_{0}^{2\pi }L_{v,sky}\left(\theta ,\phi \right)sin\theta cos\theta d\phi d\theta$$

and the scalar illuminance $E_{v,scal,hem}$ for the imaging hemisphere without cosine correction:

(2) $$E_{v,scal,hem}=\int_{0}^{\frac{\pi }{2}}\int_{0}^{2\pi }L_{v,sky}\left(\theta ,\phi \right)sin\theta d\phi d\theta$$

In the equations, $L_{v,sky}$ is the sky luminance, θ is the zenith angle and ϕ is the azimuth angle. For all-sky images, i.e., when imaging in the horizontal plane, $E_{v,hor,cos}$ is usually termed horizontal illuminance. From the three spectral channels, the correlated color temperature (CCT) can be calculated. CCT is the closest approximation to the color temperature of a perfect Planckian black body radiator for light that is not originating from an ideal Planckian radiator. A transformation from the RGB channels to CIE XYZ color space is done that then can serve as an indicator for CCT.

### 2.2. Study Sites

#### 2.2.1. Suburban Site, Ludwigsfelde, Germany

**Figure 1:**
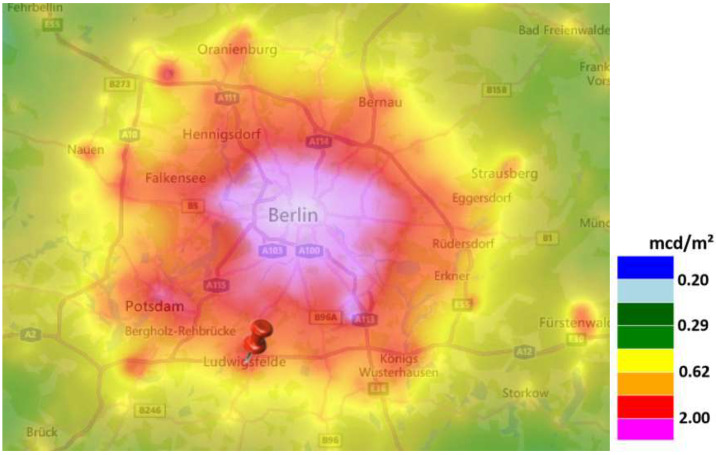
Map of the suburban site in the town of Ludwigsfelde (arrow) near Berlin, Germany using data from the world atlas of artificial night sky brightness [1] for the Berlin area (source: lightpollutionmap.info by Jurij Stare).

The first measurement location is situated about 25 km south of Berlin, Germany (population ca. 3.5 Mio) in the town of Ludwigsfelde (population 24,000) with the camera positioned at 52°17′46″ N, 13°15′41″ E. The world atlas of artificial night sky brightness [1] gives a clear-sky zenith luminance of 19.9 mag/arcsec^2^ or ca. 1.2 mcd/m^2^. The dominant source of skyglow under clear conditions is Berlin (18.1 mag/arcsec^2^ or ca. 6.2 mcd/m^2^ in city center), while another considerable skyglow source is the city of Potsdam (population 175,000; 19.4 mag/arcsec^2^ or ca. 1.8 mcd/m^2^ in city center) at 18 km distance. See Figure 1 for a map of the area using data from [1].

Measurements were performed during three nights. The first measurement was done on 11.12.2017 at 00:17 local time just after a heavy snowfall under overcast conditions. The camera was set to ISO 1600 with a shutter speed of 0.4 s. Astronomical twilight ended at 18:00 the day before and the moon (40% illumination) was 1° below the horizon with moonrise at 00:20. The second measurement was done on 12.12.2017 at 01:35 local time just after the snow melted under overcast conditions. The camera was set to ISO 3200 with a shutter speed of 0.8 s. Astronomical twilight ended at 18:00 the day before and the moon (30% illumination) was 1° above the horizon with moonrise at 01:30. The last measurement at this location was done on 07.01.2018 at 22:41 local time. That was the first moonless clear night after the first two measurements. The camera was set to ISO 3200 with a shutter speed of 10 s. Astronomical twilight ended at 18:16 and the moon (68% illumination) was 6° below the horizon with moonrise at 23:17.

#### 2.2.2. Remote Subarctic Site, Portimo, Finland

**Figure 2:**
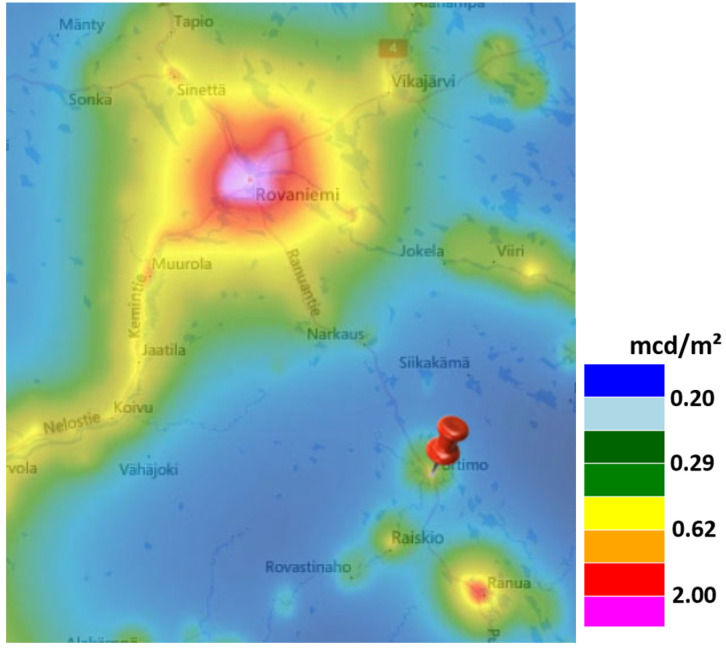
Map of the remote subarctic site in Portimo (arrow) near the city of Rovaniemi, Finland using data from the world atlas of artificial night sky brightness [1] (source: lightpollutionmap.info by Jurij Stare).

The second measurement location was on a frozen lake near the Arctic Circle next to the village of Portimo, Lapland, Finland. The measurement position was 66°05′25.4″ N, 26°20′31.5″ E. The world atlas of artificial night sky brightness [1] gives a zenith luminance of 21.3 mag/arcsec^2^ or ca. 0.3 mcd/m^2^. The next bigger settlements are the town of Ranua (population 3900) to the Southeast at ca. 20 km distance and the city of Rovaniemi (population 63,000) to the Northwest at ca. 55 km distance. See Figure 2 for a map of the area using data from [1]. Measurements were taken on two consecutive nights. The first measurement was done on 01.02.2019 at 21:17 local time just after a heavy snowfall under overcast conditions. The camera was set to ISO 3200 with a shutter speed of 5 s. Astronomical twilight ended at 18:54 and the moon was 45° below the horizon. The second measurement was done on 02.02.2019 at 22:03 local time with snow basically unaltered on the ground and clear sky conditions with a little bit of aurora activity. The camera was set to ISO 3200 with a shutter speed of 30 s. Astronomical twilight ended at 18:57 and the moon was 44° below the horizon.

#### 2.2.3. Dark Overcast Sky Reference, Saunags, Latvia

**Figure 3:**
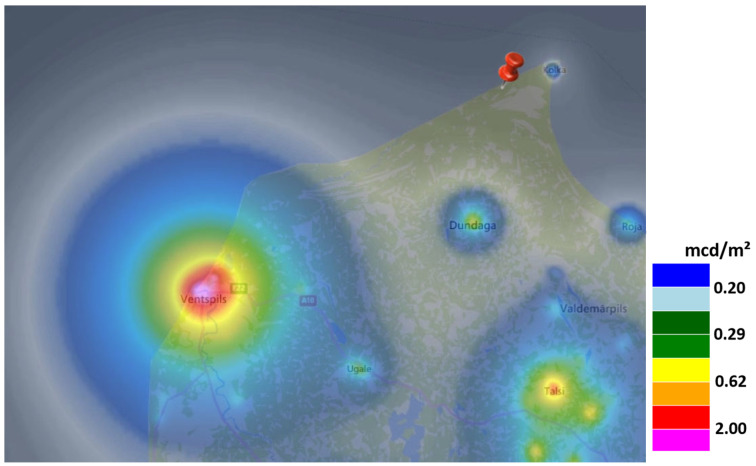
Map of the remote site in Saunags, Latvia (arrow) near the village Kolka, Latvia, on the Courland peninsula using data from the world atlas of artificial night sky brightness [1] (source: lightpollutionmap.info by Jurij Stare).

The third measurement location was on a beach near the small village of Saunags, Latvia near the tip of Cape Kolka at the Courland peninsula. The measurement position was 57°43′19.8″ N, 22°25′46.6″ E. The world atlas of artificial night sky brightness [1] gives a zenith luminance of 22.00 mag/arcsec^2^ or ca. 0.17 mcd/m^2^. The next settlements are the village of Kolka (population 800; 21.8 mag/arcsec^2^ or ca. 0.20 mcd/m^2^) to the Northeast at ca. 10 km distance and the city of Ventspils (population 38,000; 18.9 mag/arcsec^2^ or ca. 2.9 mcd/m^2^) to the Southwest at ca. 70 km distance. See Figure 3 for a map of the area using data from [1]. Data at this location was obtained on 07.09.2018 at 23:25 at an overcast night. The camera was set to ISO 6400 and a shutter speed of 120 s. Astronomical twilight ended at 22:41 local time and the moon was 15° below the horizon. As this data was obtained during a transect, there is no clear sky reference measurement, yet.

## 3. Results

### 3.1. Measurements at the Suburban Site, Ludwigsfelde, Germany

**Figure 4:**
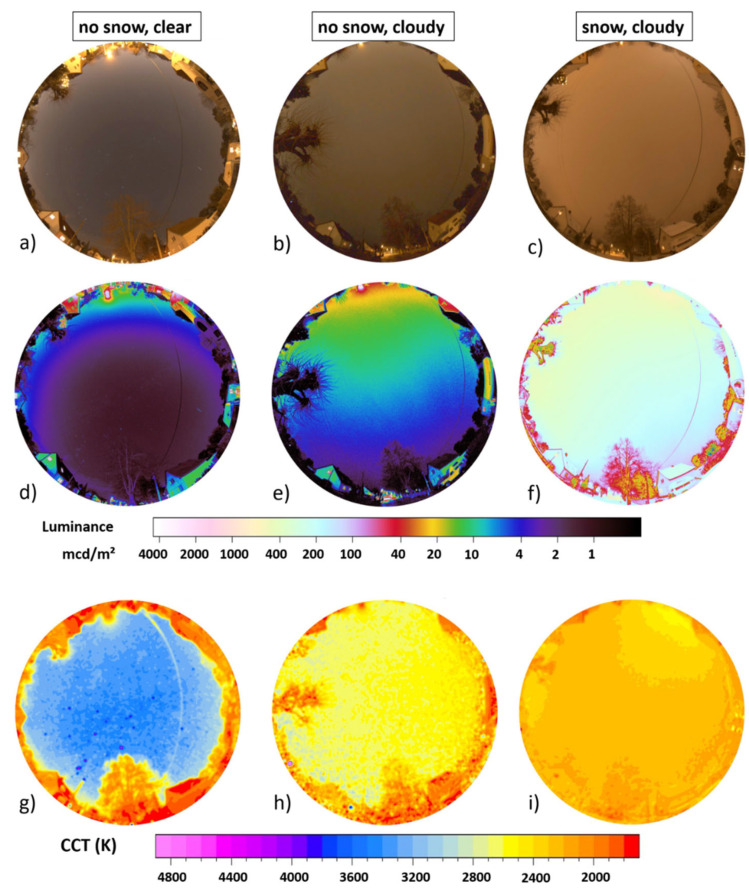
Imaging data taken during three winter nights in the suburban area in the town of Ludwigsfelde, Germany close to Berlin. Panels (**a**–**c**) show full-color RGB, panels (**d**–**f**) show luminance maps and panels (**g**–**i**) show CCT maps. The left column (**a**,**d**,**g**) shows data from a clear night without snow, middle column (**b**,**e**,**h**) shows data from an overcast night without snow and right column (**c**,**f**,**i**) shows data from an overcast night with freshly fallen snow.

Figure 4 shows the data taken during three winter nights in the suburban area in the town of Ludwigsfelde, Germany, close to the German capital Berlin (see Section 2.2.1 for details). The upper row (a,b,c) shows full-color RGB images, the middle row (d,e,f) shows luminance maps and the lower row (g,h,i) shows CCT maps. The left column (a,d,g) shows data from a clear night without snow, middle column (b,e,h) shows data from an overcast night without snow, and the right column (c,f,i) shows data from an overcast night with freshly fallen snow.

The zenith night sky brightness was 19.7 mag/arcsec^2^ or 1.3 mcd/m^2^ for the clear night without snow (Figure 4 left column), which is relatively close to the value calculated in [1], which was 19.9 mag/arcsec^2^ or 1.3 mcd/m^2^. Horizontal illuminance was 7.0 mlx and (hemispherical) scalar illuminance 24 mlx. With clouds but without snow (Figure 4 middle column), the zenith night sky brightness increases by more than a factor of 5 to 17.9 mag/arcsec^2^ or 7.3 mcd/m^2^. Horizontal illuminance was 22 mlx and (hemispherical) scalar illuminance 48 mlx. With snow and clouds (Figure 4 right column), the zenith night sky brightness increases to 14.1 mag/arcsec^2^ or 244 mcd/m^2^. This is 188 times the value without snow and clear sky and 33 times the value without snow and overcast. Horizontal illuminance was 790 mlx and (hemispherical) scalar illuminance 1500 mlx. The results, including the different amplification factors for clouds and snow, are summarized in Table 1.

**Figure 5:**
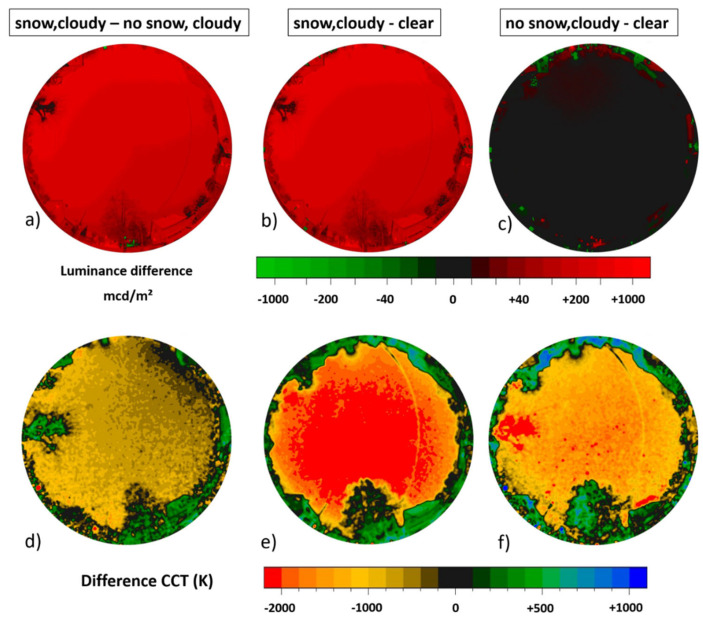
Difference in imaging data between three winter nights in the suburban area in the town of Ludwigsfelde (see Figure 4). Panels (**a**–**c**) show subtracted luminance data and panels (**d**–**f**) show subtracted CCT data. The left column (**a**,**d**) shows data obtained by subtracting imaging data of a cloudy night without snow (Figure 4e,h) from the cloudy night with snow (Figure 4f,i), the middle column (**b**,**e**) shows data obtained by subtracting the clear night without snow (Figure 4d,g) from the cloudy night with snow (Figure 4f,i) and the right column (**c**,**f**) shows data obtained by subtracting the clear night data (Figure 4d,i) from the overcast night without snow (Figure 4e,h).

Figure 5 shows the spatially resolved difference obtained by subtracting the imaging data of different nights shown in Figure 4. The upper row (a,b,c) shows difference in luminance and the lower row (d,e,f) difference in CCT. The left column (a,d) shows data obtained by subtracting the cloudy night without snow from the cloudy night with snow, the middle column (b,e) shows data obtained by subtracting the clear night without snow from the cloudy night with snow and the right column (c,f) shows data obtained by subtracting the clear night without snow from the cloudy night without snow.

### 3.2. Measurements at the Subarctic Site, Portimo, Finland

**Figure 6:**
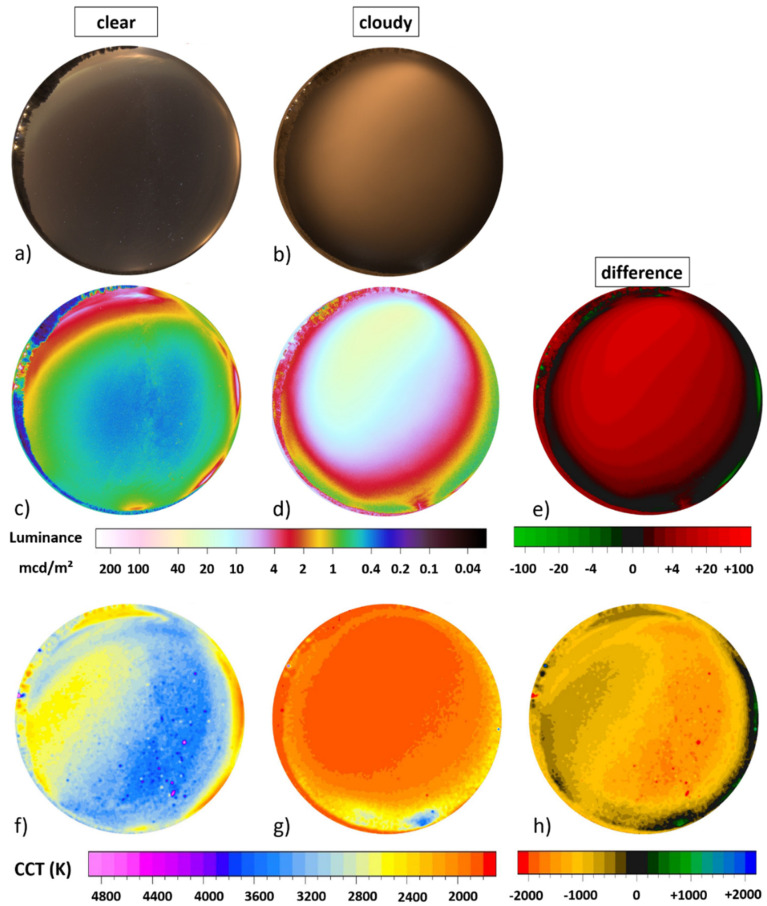
Imaging data taken during two winter nights in the subarctic village Portimo, Finland. Panels (**a**,**b**) show full-color RGB data, panels (**c**–**e**) luminance maps and panels (**f**–**h**) CCT maps. The left column (**a**,**c**,**f**) shows clear night data and the middle column (**b**,**d**,**g**) data from an overcast night. Right column (**e**,**h**) shows the difference by subtracting clear from overcast night data.

Figure 6 shows the data obtained on a frozen lake near the village of Portimo near the Arctic Circle in Finland (see Section 2.2.2 for details). The upper row (a,b) shows full-color RGB images, the middle row (c,d,e) shows luminance maps and the lower row (f,g,h) shows CCT maps. The left column (a,c,f) shows data from a clear night with snow, the middle column (b,d,g) shows data from an overcast night with snow. The difference obtained by subtracting the clear sky data from the cloudy sky data is shown in the right column (e,h).

The zenith night sky brightness was 20.9 mag/arcsec^2^ or 0.5 mcd/m^2^ for the clear night (Figure 6 left column), which is a bit higher than the value calculated in [1], which was 21.3 mag/arcsec^2^ or 0.3 mcd/m^2^. Horizontal illuminance was 2.2 mlx and (hemispherical) scalar illuminance 5.6 mlx. With clouds (Figure 4 right column), the zenith night sky brightness increases by more than a factor of 26 to 17.3 mag/arcsec^2^ or 13 mcd/m^2^. Horizontal illuminance was 34 mlx and (hemispherical) scalar illuminance 55 mlx. The results are summarized in Table 2.

### 3.3. Measurements at the Dark Overcast Sky Reference Site, Saunags, Latvia

**Figure 7:**
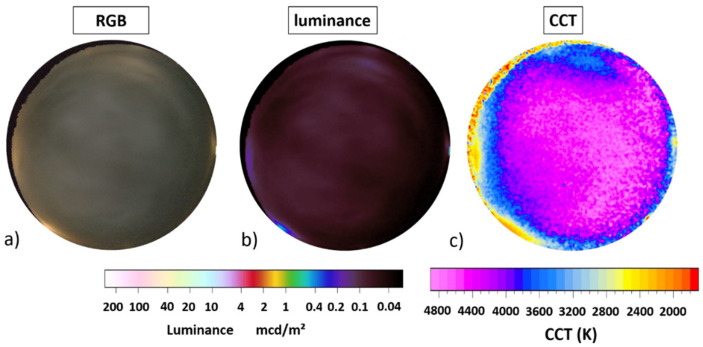
Imaging data taken during an overcast night on a beach near the village of Saunags, Latvia: (**a**) full-color RGB image, (**b**) luminance map and (**c**) CCT map. With a zenith brightness of 22.9 mag/arcsec^2^ and a horizontal illuminance of 0.22 mlx, this measurement represents the darkest site the authors have surveyed with this method so far.

Figure 7 shows the data obtained during an overcast night on a beach near the village of Saunags, near the Northern tip of the Courland peninsula, in Latvia (see Section 2.2.3 for details). Figure 7a shows the full-color RGB image, Figure 7b shows the calculated luminance map and Figure 7c shows the calculated CCT map.

The zenith night sky brightness was 22.9 mag/arcsec^2^ or 0.07 mcd/m^2^. Horizontal illuminance was 0.22 mlx and (hemispherical) scalar illuminance 0.4 mlx. To our knowledge, this is the darkest value for overcast skies that has been obtained with a calibrated camera system, but see [16,17,19] for SQM values in the same range. Results are listed in Table 3.

## 4. Discussion

The results obtained at the suburban site show a dramatic increase in night sky brightness at the zenith as well as in illuminance levels with the presence of clouds and snow. Snow amplification factors for the zenith brightness are as high as 33 for the suburban site when comparing overcast with snow and overcast without snow (Δ_3_ in Table 1). Cloud amplification factors with snow on the ground in the remote subarctic site are also on the same order of 26 (Δ_clouds_ in Table 2). We introduce the term “snowglow” for the amplification of skyglow by snow and by snow and clouds.

A commonly used comparison is to use a clear sky reference value for the night sky brightness. The currently most commonly used value in the light pollution community is 0.25 mcd/m^2^ (ca. 21.6 mags/arcsec^2^) [16]. However, sometimes a lower value of 0.17 mcd/m^2^ (ca. 22.0 mags/arcsec^2^) is used [1]. In Table 4, the measured night sky brightness values are compared with the former reference value of 0.25 mcd/m^2^ with Δ_NAT_ = L*_v,zenith_*/0.25 mcd/m^2^. The overcast sky in Finland with snow on the ground is about 50 times brighter than the clear sky reference. The German site without snow is about 30 times brighter than the clear sky reference and with snow and clouds the German site becomes almost 1000 times brighter than the clear sky reference.

However, such a comparison would only give an incomplete view, as clouds should make the sky darker without artificial light. We measured 22.9 mag/arcsec^2^ or 0.07 mcd/m^2^ with the all-sky camera in Latvia (see Section 3.3), which is in agreement with earlier results with SQMs obtained by us on Lake Stechlin, Germany [16], Ribas et al. in Montsec, Catalunya [17] and Plauchu-Frayn et al. in a dark site in Mexico [19]. Thus, it makes sense to compare (lightly polluted) cloudy skies with ALAN with a reference value obtained at a (non-light polluted) dark overcast site with illuminance values. For more details, please see the discussion in [15]. Here, a comparison between the overcast nights with snow and clouds (Germany Section 3.1 and Finland Section 3.2) is compared with the measurements in Latvia (Section 3.3). Results are listed in Table 5 also for horizontal illuminance E*_v,hor_* and scalar illuminance E*_v,hem, scal_*. When using this new reference, the amplification factors increase to ca. 190 for the Finish site with snow and clouds, ca. 100 for the German site with clouds and without snow and almost 3500 for the German site with snow and clouds.

It is further interesting to note that the CCT decreased during overcast conditions with the presence of artificial light. At the suburban site, it decreased from 3400 K for clear sky without snow to 2600 K with clouds and no snow and further to 2300 K with snow and clouds. In Finland, it decreased from 3100 K at clear skies with snow to 1900 K with clouds and snow. On the other hand, the CCT remained relatively high at the dark summer sky reference site in Latvia with 4500 K. Unfortunately, no clear sky reference for this site is available, yet.

Another striking result is the illuminance values obtained at the German site with snow and clouds: horizontal illuminance E*_v,hor_* ≈ 0.79 lx and scalar illuminance E*_v,hem, scal_* ≈ 1.5 lx that are also on the order of 3600 times and 3800 times higher than the summer dark site reference values measured in Latvia. Furthermore, the measured value for horizontal illuminance is more than a factor of 2 higher than the highest possible value of 0.32 lx for a full moon at zenith (which is only possible at the equator). Please note that a “typical” full moon in the latitude of Berlin is rather of the order of 0.1 lx–0.2 lx and that such values are only reached at a short time interval.

Unfortunately, the dataset presented here is not perfect. A clear sky reference (without snow) and measurements with snow cover in Latvia, a clear sky measurement with snow in Germany and measurements without snow in Finland would be a nice addition. For further studies, a comprehensive dataset using all-sky [14,15,16,18,20,21] or even full-sphere imagery with DSLR cameras [28] is advised. Ideally, imaging devices should be used for long-term monitoring, because right now they mainly complement measurements of single channel devices such as SQMs [16]. Long-term data from networks can provide a breadth of information as shown with SQM data [26]. Furthermore, different type of clouds and cloud elevations [17] are worth studying. An interesting point is the dependence of cloud reflectivity by optical thickness of clouds as modelled by Garstang [31].

## 5. Conclusions

In conclusion, the combined amplification of skyglow by clouds and snow can reach very high values even in a suburban setting and be significant in a remote setting. To the authors’ knowledge, this is the first time that all-sky DSLR photometry is used to investigate the problem that we term “snowglow”. With the imaging data, we can compare zenith brightness but also illuminance values derived from the hemispherical data. The results are rather alarming, as amplification factors of up to 3500 times higher than the dark overcast sky reference were determined at the suburban site. It is again pointed out, that for clear sky conditions, the suburban site appears only polluted on a medium level: the summer Milky Way is visible on clear nights and the winter Milky Way occasionally at very cold and therefore clear nights with low humidity. Nevertheless, the horizontal illuminance from snowglow (with snow cover and clouds) exceeds the maximum value of a full moon by more than a factor of 2, which is rated very light polluted. Such a high degree of light pollution could be a relevant stressor from an ecological perspective as it can directly impact nocturnal organisms, mediates trophic and social interactions, and eliminates monthly variation in moon light levels, which are often necessary to trigger circalunar rhythms and shape life-history strategies [32,33,34,35].

A solution for the amplification from “snowglow” regarding lighting technology would be that illuminance levels should be reduced with the presence of snow, which is possible with dimmable, adaptive, smart lighting technology readily commercially available today. 

## Figures and Tables

**Table 1:** List of parameters measured at zenith at the suburban site in Ludwigsfelde, Germany during three winter nights with different cloud and snow cover.

	Clear, No Snow	Overcast, No Snow	Overcast, Snow	Δ_1 (*)_	Δ_2 (**)_	Δ_3 (***)_
L*_v,zenith_* (mcd/m^2^)	1.3	7.3	244	5.6	188	33
E*_v,hor_* (mlx)	7	22	790	3	113	36
E*_v,hem, scal._* (mlx)	24	48	1500	2	63	31
CCT_zen_ (K)	3400	2600	2300			

(*) cloud amplification: Δ_1_ = overcast, no snow/clear, no snow; (**) cloud and snow amplification Δ_2_ = overcast, snow/clear; (***) snow amplification: Δ_3_ = overcast, snow/overcast, no snow.

**Table 2:** List of parameters measured at zenith at the subarctic site in Portimo, Finland on two winter nights with different cloud cover and the same snow cover.

	Clear, Snow	Overcast, Snow	Δ_clouds (*)_
L*_v,zenith_* (mcd/m^2^)	0.5	13	26
E*_v,hor_* (mlx)	2.2	34	15
E*_v,hem, scal._* (mlx)	5.6	55	10
CCT_zen_ (K)	3100	1900	

(*) cloud amplification: Δ_clouds_ = overcast, snow/clear, snow.

**Table 3:** List of parameters measured at zenith at the dark reference site in Latvia during a summer night under fully overcast conditions.

	Overcast Summer
L*_v,zenith_* (mcd/m^2^)	0.07
E*_v,hor_* (mlx)	0.22
E*_v,hem, scal._* (mlx)	0.40
CCT_zen_ (K)	4500

**Table 4:** Comparing the parameters of the winter overcast nights in Finland and Germany to the commonly used average clear sky night sky brightness of 0.25 mcd/m^2^ (ca. 21.6 mags/arcsec^2^).

	Finland Overcast, Snow	Δ_NAT (*)_	Germany Overcast, No Snow	Δ_NAT (*)_	Germany Overcast, Snow	Δ_NAT (*)_
L*_v,zenith_* (mcd/m^2^)	13	52	7.3	29	244	980

(*) Δ_NAT_ = L*_v,zenith_*/0.25 mcd/m^2^.

**Table 5:** Comparing the parameters of the winter overcast nights in Finland and Germany to the summer reference measured in Latvia and calculating the amplification using the summer reference.

	Latvia Overcast Reference	Finland Overcast, Snow	Δ_FI, CL, SN (*)_	Germany Overcast, No Snow	Δ_GER, CL (**)_	Germany Overcast, Snow	Δ_GER, CL, SN (***)_
L*_v,zenith_* (mcd/m^2^)	0.07	13	185	7.3	104	244	3490
E*_v,hor_* (mlx)	0.22	34	154	22	100	790	3590
E*_v,hem, scal._* (mlx)	0.40	55	138	48	120	1500	3750
CCT_zen_ (K)	4500	1900		2600		2300	

(*) Δ_FI, CL, SN_ = overcast, snow (Finland)/overcast summer (Latvia), (**) Δ_GER, CL_ = overcast, no snow (Germany)/overcast summer (Latvia), (***) Δ_GER, CL, SN_ = overcast, snow (Germany)/overcast summer (Latvia).
